# When the night becomes a nightmare

**DOI:** 10.5935/0103-507X.20220017-en

**Published:** 2022

**Authors:** Flavio Eduardo Nacul, Andre Volschan

**Affiliations:** 1 Hospital Pró-Cardíaco - Rio de Janeiro (RJ), Brazil.

Macbeth^(1)^ describes sleep as the “balm of hurt minds, great nature’s second course, chief nourisher in life’s feast, a soothing bath after a day of hard work, and the main course of a feast”. Although scientists are still working to identify and clarify all of the functions of sleep, decades of studies have confirmed that sleep is essential for survival and healthy functioning, as well as optimal physical and cognitive performance.

The connections between sleep disruption and disease have become more firmly established over time. It is well known that poor quality sleep can have significant adverse consequences for hospitalized patients, prompting emotional distress and *delirium*.^(2)^ Several studies have shown that patients in the intensive care unit exhibit significant alterations in highly fragmented sleep architecture, with prolonged sleep latencies and poor efficiency.^(3)^

Although rest is a goal of patients when they are in the hospital, most of them develop a period of acute sleep deprivation due to environmental, medical, and patient-specific factors, and the need for adequate rest is very difficult to obtain during a hospital stay. Several factors related to sleep deprivation in hospitalized patients include noise, light, awakenings by medical staff, and factors related to the patients, such as pain, stress, and anxiety.^(4,5)^

In general, interventions to improve sleep in hospitalized adults can be nonpharmacologic or pharmacologic, and it is generally recommended that nonpharmacologic interventions be the first line of therapy. In the event that aid with pharmacologic sleep is needed, the choice of drug should be customized based on the patient profile.

Staying awake at night, until dawn or being woken up several times in the middle of the night are not desirable and unacceptable. In this scenario, the best choice would be to let patients drift into deep sleep. After being hospitalized at different periods and listening to our patients, both authors observed that a night in the hospital can be a nightmare, especially when you cannot sleep well. Because we believe that getting a better night’s sleep in the hospital can improve healing, we have undertaken a new initiative to improve the quality and quantity of sleep of all hospitalized patients in our hospital. Specifically, we have created a multidisciplinary working group with the shared purpose of developing better and more effective solutions to promote sleep in hospital settings. Our “sleep ‘team’ published a hospital policy that includes a quiet time between 11 PM and 6 AM and interventions such as improving staff awareness of noise, reducing night light levels, and changing the timing of hospital routine delivery, including medication administration, laboratory tests, and procedures, when possible. Additionally, it includes offering earplugs and masks and avoiding scheduling maintenance, housekeeping, nutrition pick ups, and noisy procedures during quiet time. It is important to note that quiet time is not a no-care time. It simply allows for the performance of patient care in a quieter and less disruptive manner during these hours. Studies have shown that the implementation of a quiet time has positive results.^(6)^

Despite several scientific findings and increased awareness, the importance of sleep optimization is still relatively low on the list of priorities in hospitals.^(7)^ Overall evidence about interventions that could be performed to improve perceived sleep quality in hospitalized patients was found to be positive. In addition, the quantity and quality of sleep play an essential role in a patient’s health and demand consideration in any treatment plan.^(8)^ Reductions of the adverse effects of noise, light, uncomfortable bedding, intrusive observations, anxiety, and pain, together with attention to specific sleep needs and the monitoring of sleep quality, are steps that would help in addressing the issue and can potentially improve patient outcomes.^(9)^ It is also clear that any successful solution will be multifactorial and require the involvement of many stakeholders, including leadership, architects, suppliers, environmental services, laboratories, nurses, and clinicians.^(10)^ All of the members of the treatment team can contribute to assessing and optimizing sleep for hospitalized patients. The time for action has arrived. A good laugh and a long sleep are the best cures in the doctor’s book (Irish Proverb).
